# *Ni-kshay Poshan Yojana*: receipt and utilization among persons with TB notified under the National TB Elimination Program in India, 2022

**DOI:** 10.1080/16549716.2024.2363300

**Published:** 2024-07-22

**Authors:** Kathiresan Jeyashree, Jeromie W V Thangaraj, Devika Shanmugasundaram, G Sri Lakshmi Priya, Sumit Pandey, Venkateshprabhu Janagaraj, Prema Shanmugasundaram, Sumitha Ts, Sabarinathan Ramasamy, Joshua Chadwick, Sivavallinathan Arunachalam, Rahul Sharma, Vaibhav Shah, Aniket Chowdhury, Swati Iyer, Raghuram Rao, Sanjay K Mattoo, Manoj V Murhekar, NPY Evaluation Group

**Affiliations:** a Division of Epidemiology and Biostatistics, ICMR-National Institute of Epidemiology, Chennai, India; b TB Support Network, WHO Country Office for India, New Delhi, India; c Central TB Division, Ministry of Health and Family Welfare, New Delhi, India

**Keywords:** Direct benefit transfer, nutrition, delay, pattern of utilization, treatment outcome

## Abstract

**Background:**

*Ni-kshay Poshan Yojana* (NPY), a direct benefit transfer scheme under the National Tuberculosis Elimination Program (NTEP) in India, provides a monthly benefit of INR500 for nutritional support of persons with TB (PwTB).

**Objectives:**

To determine the proportion of PwTB receiving atleast one NPY instalment and pattern of utilisation; to ascertain factors associated with NPY non-receipt and association of NPY receipt with TB treatment outcome.

**Methods:**

In our cross-sectional study, we used multi-stage sampling to select PwTB whose treatment outcome was declared between May 2022 and February 2023. A cluster-adjusted, generalized linear model was used to identify factors associated with the non-receipt of NPY and determine association between NPY receipt and TB treatment outcome.

**Results:**

Among 3201 PwTB, 2888 (92.7%; 95% CI 89.8%, 94.8%) had received at least one NPY instalment, and 1903 (64.2%; 95% CI 58.9%, 69.2%) self-reported receipt of benefit. The median (IQR) time to receipt of first instalment was 105 (60,174) days. Non-receipt was significantly higher among PwTB from states with low TB score (aPR = 2.34; 95%CI 1.51, 3.62), who do not have bank account (aPR = 2.48; 95%CI 1.93, 3.19) and with unknown/missing diabetic status (aPR = 1.69; 95%CI 1.11, 2.55). Unfavorable treatment outcomes were associated with non-receipt of NPY (aPR 4.93; 95%CI 3.61,6.75) after adjusting for potential confounders.

**Conclusion:**

Majority of the PwTB received atleast one NPY instalment, but they experience significant delays. Most of the recipients utilised NPY for nutrition. Longitudinal follow-up studies are required to study the impact of NPY on treatment outcomes.

## Paper context

- **Main findings:** Five years since implementation, almost ninety percent of persons with tuberculosis in India had received at least one benefit under *Ni-kshay Poshan Yojana*, most of whom utilise it for nutrition.
- **Added knowledge:** Persons with tuberculosis in India experience a median delay of over three months to receive the first instalment of the benefit.
- **Global health impact for policy and action:** Ensuring timely credit of benefits is crucial to enable its utilisation by persons with tuberculosis for better nutrition and favourable treatment outcomes.

## Background

Undernutrition is a significant risk factor for incidence of tuberculosis (TB) infection [1,2]. Over 25% of TB incidence in high TB burden countries is attributed to undernutrition [3–5], which is much higher than other risk factors like smoking (11%), diabetes (9%), and Human Immunodeficiency Virus (HIV) (5%) [6,7]. Undernutrition is also linked to TB disease severity and poor treatment outcomes [6,8] including increased mortality [9] by compromising immunity, influencing the pharmacodynamics, pharmacokinetics, tolerance, and adherence of the anti-tuberculosis treatment (ATT) [10].

Nutritional support is recommended as an integral part of TB management by WHO [11]. Nutrition supplementation interventions have effectively reduced TB incidence among household contacts [12], decreased mortality [12,13] and improved treatment outcomes [14]. Cash transfer schemes have been shown to improve treatment outcome in PwTB [15–18]. Non-receipt of direct benefit transfer (DBT) (during treatment) has been reported to be associated with death, loss to follow-up and treatment failure [15].

India suffers from high prevalence of undernutrition among PwTB [19] while also being one of the highest TB burden countries in the world. The Government of India has made a commitment to achieve the sustainable development goal end TB targets, by 2025. Under the ‘National strategic plan for TB elimination 2017–2025’, there are DBT initiatives that target all patients, specific vulnerable groups of patients like tribals, treatment providers in the private sector or treatment supporters like the Accredited Social Health Activist (ASHA) [20]. One of the DBT initiatives is the *‘Ni-kshay Poshan Yojana’* (NPY), which was launched in 1 April 2018 [21,22] to provide nutritional support to all PwTB. Under NPY, all notified PwTB receive INR 500 per month (~US$7) throughout the period of ATT [23,24]. The benefit is credited directly to the bank or post office accounts of the PwTB [20] through Public Financial Management System (PFMS).

The NPY is expected to enable PwTB to spend on their additional nutritional requirements and thus improve their nutritional status [25] and further increase the probability of favourable TB treatment outcomes. Additionally, it can also support the TB affected households in meeting the additional costs due to TB diagnosis and care and acts as an incentive for treatment adherence [15]. Previous evaluations of NPY conducted in smaller geographical regions of India had reported low coverage of NPY with longer delays [23,26–29] and documented early implementation challenges.

Entering the sixth year of its implementation, there has been no nationwide evaluation of NPY implementation and utilisation or assessment of its impact on TB treatment outcomes yet. Such evidence is required to provide vital insights to the national TB program to ensure optimal implementation of NPY. We determined the a) proportion of PwTB who had received at least one instalment, b) time to receive the first instalment, c) pattern of utilisation of the benefit, and d) impact of NPY receipt on treatment outcome.

## Methods

### Study design

Cross-sectional study using primary data from PwTB and secondary data from *Ni-kshay*, the case based real time data management system in National TB Elimination Program (NTEP).

### Study setting

India is administratively divided into 28 states and eight Union Territories, which are further divided into administrative units called districts.

Under the NTEP, the district TB centres monitor the programme implementation through a network of sub-district level tuberculosis units (TUs) consisting of Peripheral Health Institutions (PHI) from both public and private sectors. All states and Union Territories of India have implemented NPY. On diagnosis, NTEP health workers collect the bank details of PwTB and the state/district level program managers validate it before forwarding them to the PFMS. The benefit will be credited upon validation of the bank or post office account details by the PFMS. The first two NPY instalments are credited together as INR 1000 (US$ 12.1) soon after diagnosis. Subsequently, advance monthly benefits of INR 500 (~US$7) are paid until their TB treatment outcome is declared.

We conducted this study in 30 districts across nine states in India namely, Bihar, Delhi, Rajasthan, Tamil Nadu, Telangana, Uttarakhand, Gujarat, Meghalaya, and Odisha under the NTEP programmatic setting.

### Study population

We included PwTB aged ≥18 years, who were notified under the NTEP and whose TB treatment outcome had been declared between May 2022 and February 2023.

### Sampling and sample size

We divided the states into three strata based on TB score (a composite score measuring NTEP performance and TB burden): high, medium, and low [30] (Supplementary table S1). From each stratum, we selected three states by simple random sampling and from the nine selected states, we selected thirty NTEP districts based on probability proportionate to size (TB notification) sampling. From each of the selected NTEP districts, we selected two TUs and from each TU we selected two/three PHIs using probability proportionate to size (TB notification) sampling.

Assuming that 50% of all notified PwTB would have received NPY benefit [23], we calculated a minimum required sample size of 960 persons (rounded up to 1000 per stratum) allowing a 5% alpha error, design effect of 2, absolute precision of 5% and 20% non-response. We obtained the list of all PwTB (*N* = 17608) in selected PHIs (*N* = 98) and randomly selected 25 to 30 PwTB per PHI.

### Data collection

We collected data by in-person interviews from the selected PwTB or family members of deceased PwTB in their respective households using a structured electronic questionnaire on Open Data Kit (ODK) platform. We obtained PwTB demographic and clinical characteristics, and treatment outcome from current notification register (*Ni-kshay*), number of instalments and amount credited per instalment from NPY beneficiary register, and the dates of credit of each instalment from DBT Turn-around time (TAT) indicator register (*Ni-kshay*). We defined the TB treatment outcome as favourable if the documented outcome was either cured or completed.

### Data analysis

The primary data were merged with the secondary data using the episode ID (*Ni-kshay* identifier). The proportion of PwTB who received at least one NPY instalment was estimated from the self-reported primary data and secondary data (*Ni-kshay*). We calculated cluster adjusted and weighted proportion of receiving at least one NPY instalment along with 95% Confidence Interval (CI) for each stratum using design weight and adjusting for non-response. We estimated the overall proportion of receipt of at least one NPY instalment based on stratum proportions. The time to receipt was calculated as the duration between the date of diagnosis and date of receipt of the first instalment. A cluster-adjusted, generalized linear model with the Poisson family and log link was used to identify factors associated with the non-receipt of NPY and to look for association between NPY receipt and unfavourable treatment outcome. To determine the association between receipt of NPY and treatment outcome, we defined receipt as PwTB who had received the first instalment before declaration of treatment outcome. Receiving the first instalment of NPY after declaration of treatment outcome was counted as non-receipt of NPY. Statistical analysis was done using Stata V.17.0 and R v.4.3.2.

### Ethics and consent

The Institutional Human Ethics Committee (IHEC) of Indian Council of Medical Research – National Institute of Epidemiology (ICMR-NIE) (NIE/IHEC/202201–13) approved the study, and a non-disclosure agreement was signed between ICMR-NIE and Central TB Division for sharing the data based on the study requirements. We obtained administrative approvals from the state and district TB centres. We received informed written consent from all the study participants or family members (in case of deceased PwTB) after sharing the participant information sheet in the regional language.

## Results

### Sociodemographic and clinical characteristics

Of the 3201 PwTB, 1123 (35.1%) were from the low TB score stratum, 1975 (61.7%) were male, and 1381 (43.1%) were economically inactive. About 2581 (80.6%) had their own bank account. The median (IQR) monthly household income before TB was US$ 181.7 (116.3, 278.6). Medical insurance was accessible to 36.7% (*n* = 517) of the PwTB.

About 2698 (84.3%) PwTB were newly notified, and 2911 (90.9%) were notified from the public sector. There were 2421 (75.6%) persons with pulmonary TB and 3128 (97.7%) with drug-sensitive tuberculosis (DSTB). Thirty-two (1.0%) PwTB were HIV reactive and 307 (9.6%) were recorded as having diabetes mellitus. Overall, 248 (7.7%) PwTB had experienced unfavourable treatment outcomes (Table 1).

**Table 1.** Stratum wise socio-demographic and clinical characteristics of PwTB in India, 2022 (N = 3201).

Characteristics	Overall n (%)	Low TB Score n (%)	Medium TB Score n (%)	High TB Score n (%)
**Total**	**N = 3201**	**N = 1123 (35.1%)**	**N = 1028 (32.1%)**	**N = 1050 (32.8%)**
**Age (in Years)**				
≥18 to ≤ 59	2607 (81.4)	931 (82.9)	809 (78.7)	867 (82.6)
≥60	594 (18.6)	192 (17.1)	219 (21.3)	183 (17.4)
**Gender**				
Male	1975 (61.7)	661 (58.9)	643 (62.5)	671 (63.9)
Female	1226 (38.3)	462 (41.1)	385 (37.5)	379 (36.1)
**Education**				
Cannot read or write	752 (23.5)	297 (26.4)	194 (18.9)	261 (24.9)
Literate without formal schooling	408 (12.8)	161 (14.3)	108 (10.5)	139 (13.2)
Any formal schooling	1604 (50.1)	493 (43.9)	551 (53.6)	560 (53.3)
Any college education	437 (13.6)	172 (15.3)	175 (17.0)	90 (8.6)
**Occupation**^**†**^				
Self-employed	424 (13.3)	112 (10.0)	138 (13.4)	174 (16.6)
Employed	1396 (43.6)	452 (40.2)	440 (42.8)	504 (48.0)
Economically inactive	1381 (43.1)	559 (49.8)	450 (43.8)	372 (35.4)
**Bank or Post office account**				
Yes, self	2581 (80.6)	1029 (91.6)	839 (81.6)	713 (67.9)
Yes, relative’s/others’	409 (12.8)	41 (3.7)	143 (13.9)	225 (21.4)
No	138 (4.3)	39 (3.5)	21 (2.0)	78 (7.4)
Do not know	73 (2.3)	14 (1.2)	25 (2.4)	34 (3.2)
**Monthly household income before TB** Median (IQR) US $*	181.7 (116.3, 278.6)	181.7 (121.1, 278.6)	181.7 (121.1, 302.8)	157.4 (102.9, 247.1)
**INR**	15000 (9600, 23000)	15000 (10000, 23000)	15000 (10000, 25000)	13000 (8500, 20400)
**Monthly income of person before TB** Median (IQR) US $**	96.9 (60.6, 145.3)	109.0 (72.7, 145.3)	121.1 (72.7, 181.7)	84.8 (60.6, 121.1)
**INR**	8000 (5000, 12000)	9000 (6000, 12000)	10000 (6000, 15000)	7000 (5000, 10000)
**Notifying sector**				
Public	2911 (90.9)	954 (84.9)	947 (92.1)	1010 (96.2)
Private	290 (9.1)	169 (15.1)	81 (7.9)	40 (3.8)
**Type of patient**				
New	2698 (84.3)	953 (84.9)	896 (87.2)	849 (80.9)
PMDT	72 (2.3)	24 (2.1)	21 (2.0)	27 (2.6)
Retreatment	430 (13.4)	145 (12.9)	111(10.8)	174 (16.6)
Unknown/missing	1 (0.03)	1 (0.1)	0 (0.0)	0 (0.0)
**Site of disease**				
Extrapulmonary	763 (23.8)	322 (28.7)	227 (22.1)	214 (20.4)
Pulmonary	2421 (75.6)	797 (71.0)	797 (77.5)	827 (78.8)
Unknown/missing	17 (0.5)	4 (0.3)	4 (0.4)	9 (0.8)
**Drug type**				
DSTB	3128 (97.7)	1098 (97.7)	1007 (98.0)	1023 (97.4)
DRTB	72 (2.2)	24 (2.2)	21 (2.0)	27 (2.6)
Unknown/Missing	1 (0.1)	1 (0.1)	0 (0.0)	0 (0.0)
**HIV**				
Reactive	32 (1.0)	4 (0.4)	18 (1.7)	10 (0.9)
Non-reactive	3116 (97.3)	1104 (98.3)	1009 (98.2)	1003 (95.5)
Unknown/missing	53 (1.7)	15 (1.3)	1 (0.1)	37 (3.5)
**Diabetes**				
Yes	307 (9.6)	78 (6.9)	146 (14.2)	83 (7.9)
No	2782 (86.9)	1003 (89.3)	868 (84.4)	911 (86.8)
Unknown/missing	112 (3.5)	42 (3.7)	14 (1.4)	56 (5.3)
**Treatment outcome**				
Favourable	2953 (92.3)	1064 (94.7)	936 (91.1)	953 (90.8)
Unfavourable	248 (7.7)	59 (5.3)	92 (8.9)	97 (9.2)

^†^Self-employed (business/farm/shop); employed (regular employee government/regular employee private/temporary employee (government and private)/skilled worker/daily wage earner); economically inactive (unemployed/homemaker/retired/pensioner/student); *n = 3185, **n = 1996.

Abbreviations: PMDT, Programmatic Management of Drug Resistant Tuberculosis; DRTB, Drug-resistantuberculosis; DSTB, Drug-sensitive tuberculosis; HIV, Human Immunodeficiency Virus

### *Ni-kshay Poshan Yojana* receipt

**Figure 1.**
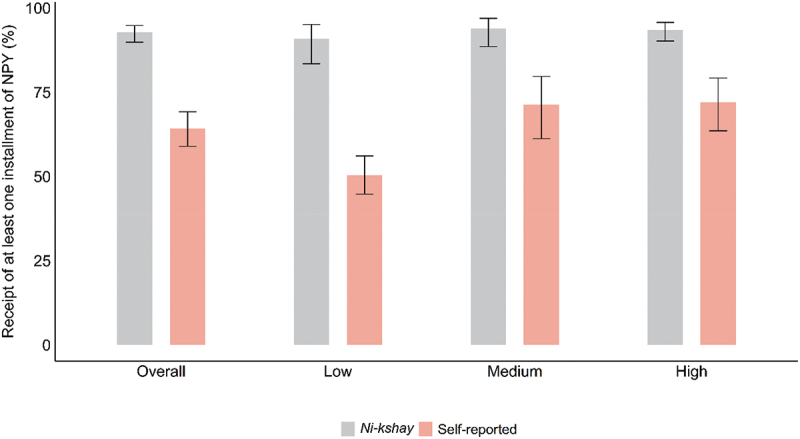
Stratum wise receipt of *Ni-kshay Poshan Yojana* among PwTB, India, 2022 (N = 3201).

As per *Ni-kshay*, the first NPY instalment was credited to 2888 (92.7%; 95% CI 89.8%, 94.8%) PwTB of whom, 65.9% were aware of NPY receipt. Overall, when interviewed, 1903 (64.2%; 95% CI 58.9%, 69.2%) self-reported the receipt of at least one NPY instalment. The proportion of NPY receipt ranged from 90.8% (95% CI; 83.4%, 95.1%) in the low TB score stratum to 93.9% (95% CI; 88.5%, 96.9%) in the medium TB score stratum (Figure 1). The median (range) amount received by NPY recipients was INR 3000 (500,10500). Of the 2953 PwTB with favourable treatment outcomes, 2221 (75.2%) had received at least INR 3000 as benefit.

### Time to receipt of first Ni-kshay Poshan Yojana instalment

The median (IQR) time to receipt of first instalment was 105 (60,174) days. Overall, 1212 (42%) had received the first NPY instalment within two months of diagnosis, while 773 (24.1%) PwTB had received it after declaration of treatment outcome (Supplementary table S2).

### Pattern of utilization of Ni-kshay Poshan Yojana

Of the 1903 PwTB who reported receipt of at least one NPY instalment, 1510 (79.4%) had completely spent the money, and 1400 (88.8%) (among partially and completely spent) had spent it on their nutritional requirements. About 86.6% (*n* = 1648) self-reported that the NPY incentive was insufficient to meet their nutritional requirements (Table 2).

**Table 2.** Pattern of utilization of *Ni-kshay Poshan Yojana* instalments self-reported by the PwTB, India, 2022 (*N* = 1903).

Characteristics	Overall n (%)	Low TB Score n (%)	Medium TB Score n (%)	High TB Score n (%)
Total*	**n = 1903**	**n = 546 (28.7%)**	**n = 681 (35.8%)**	**n = 676 (35.5%)**
**Utilized NPY benefit**				
Partially	67 (3.5)	7 (1.3)	13 (1.9)	47 (6.9)
Completely	1510 (79.4)	428 (78.4)	569 (83.5)	513 (75.9)
Saved for future	180 (9.5)	73 (13.4)	46 (6.7)	61 (9.0)
Do not know	146 (7.7)	38 (7.0)	53 (7.8)	55 (8.1)
**Expenditure of NPY benefit**^#^				
Nutritional requirements for oneself	1400 (88.8)	414 (95.2)	507 (87.1)	479 (85.5)
Nutritional requirements of the family	259 (16.4)	41 (9.4)	126 (21.7)	92 (16.4)
Family (excluding food and nutrition)	61 (3.9)	7 (1.6)	24 (4.1)	30 (5.4)
Repayment of loan/others	22 (1.4)	2 (0.5)	13 (2.2)	7 (1.3)
**NPY incentive insufficient**	1648 (86.6)	486 (89.0)	586 (86.1)	576 (85.2)

*Received at least one NPY instalment, ^#^Partially and completely spent the NPY instalments.

### Factors associated with non-receipt of Ni-kshay Poshan Yojana instalment

PwTB from the low TB score stratum (aPR = 2.34; 95% CI 1.51, 3.62), PwTB who do not have their own bank accounts (aPR = 2.48; 95% CI 1.93, 3.19) and with unknown/missing diabetic status (aPR = 1.69; 95% CI 1.11, 2.55) were significantly (*p* < 0.05) associated with non-receipt of at least one NPY instalment (Table 3). There was no difference in the proportion of PwTB receiving NPY benefits across income quintiles.

**Table 3.** Factors associated with non-receipt of at least one *Ni-kshay Poshan Yojana* instalment (based on *Ni-kshay*) among PwTB, India,2022 (*N* = 3201).

Characteristics	N (%)	Non-recipients of NPY N = 313 (9.8%)	Unadjusted PR* (95% CI)	Adjusted PR (95% CI)
**Stratum**				
Low	1123 (35.1)	172 (15.3)	1.71 (1.17, 2.50)	2.34 (1.51, 3.62)
Medium	1028 (32.1)	47 (4.6)	0.51 (0.30, 0.86)	0.59 (0.34, 1.02)
High	1050 (32.8)	94 (8.9)	1	1
**Age (in Years)**				
≥18 to ≤ 59	2607 (81.4)	261 (10.0)	1.14 (0.86, 1.52)	-
≥60	594 (18.6)	52 (8.7)	1	-
**Gender**				
Male	1975 (61.7)	199 (10.1)	1.08 (0.86, 1.37)	-
Female	1226 (38.3)	114 (9.3)	1	-
**Education**				
Cannot read or write	752 (23.5)	81 (10.8)	1.34 (0.87, 2.07)	1.08 (0.70, 1.67)
Literate without formal schooling	408 (12.8)	38 (9.3)	1.16 (0.74,1.82)	0.98 (0.63, 1.52)
Any formal schooling	1604 (50.1)	159 (9.9)	1.24 (0.85, 1.81)	1.16 (0.79, 1.70)
Any college education	437 (13.6)	35 (8.0)	1	1
**Occupation**^†^				
Self employed	424 (13.3)	37 (8.7)	0.90 (0.65, 1.25)	-
Employed	1396 (43.6)	135 (9.7)	1	-
Economically inactive	1381 (43.1)	141 (10.2)	1.06 (0.85, 1.31)	-
**Bank account**				
Yes	2581 (80.6)	213 (8.2)	1	1
No	620 (19.4)	100 (16.1)	1.95 (1.54, 2.48)	2.48 (1.93, 3.19)
**Monthly household Income before TB (quintiles)**^#^				
1st	643 (20.1)	46 (7.1)	0.79 (0.53, 1.18)	-
2nd	703 (22.0)	76 (10.8)	1.19 (0.86, 1.64)	-
3rd	601 (18.8)	66 (11.0)	1.21 (0.86, 1.69)	-
4th	601 (18.8)	62 (10.3)	1.13 (0.85, 1.52)	-
5th	637 (19.9)	58 (9.1)	1	-
Unknown/Missing	16 (0.5)	5 (31.2)	-	-
**Notifying sector**				
Public	2911 (90.9)	273 (9.4)	1	1
Private	290 (9.1)	40 (13.8)	1.47 (0.94, 2.30)	1.35 (0.79, 2.29)
**Site of disease**^**@**^				
Extra pulmonary	763 (23.8)	60 (7.9)	0.79 (0.60, 1.04)	0.75 (0.56, 1.01)
Pulmonary	2421 (75.6)	240 (9.9)	1	1
Unknown/missing	17 (0.5)	13 (76.5)	-	-
**Drug type**				
DSTB	3128 (97.7)	305 (9.7)	-	-
DRTB	72 (2.2)	7 (9.7)	-	-
Unknown/missing	1 (0.03)	1 (100.0)	-	-
**HIV**				
Reactive	32 (1.0)	2 (6.2)	-	-
Non-reactive	3116 (97.3)	302 (9.7)	-	-
Unknown/missing	53 (1.7)	9 (17.0)	-	-
**Diabetes**				
Diabetic	307 (9.6)	26 (8.5)	0.90 (0.61, 1.33)	1.12 (0.80, 1.59)
Non-diabetic	2782 (86.9)	261 (9.4)	1	1
Unknown/missing	112 (3.5)	26 (23.2)	2.47 (1.73, 3.54)	1.69 (1.11, 2.55)

^**†**^Self-employed (business/farm/shop); employed (regular employee government/regular employee private/temporary employee (government and private)/skilled worker/daily wage earner); economically inactive (Uunemployed/homemaker/retired/pensioner/student).

*For adjusted PR, *p* value of 0.2 is considered for statistical significance, 1 – reference category.

#*N* = 3185; @N = 3184.

Abbreviations: DSTB, Drug-sensitive Tuberculosis; DRTB, Drug-resistant Tuberculosis; HIV, Human Immunodeficiency Virus.

### Ni-kshay Poshan Yojana receipt and TB treatment outcome

Sixty six (3.1%) PwTB who had received first instalment of NPY and 182 (16.8%) PwTB who had not received NPY experienced unfavourable treatment outcome. Non-receipt of NPY (aPR 4.93; 95% CI 3.61, 6.75) was significantly associated with suffering an unfavourable treatment outcome after adjusting potential confounders namely socio-demographic and clinical characteristics (Table 4).

**Table 4.** Association of non-receipt of at least one *Ni-kshay Poshan Yojana* instalment with unfavourable treatment outcomes in PwTB, India, 2022 (*N* = 3201).

Characteristics	N (%)	Unfavourable treatment outcome N = 248 (7.8%)	Unadjusted PR (95% CI)*	Adjusted PR (95% CI)
**NPY receipt**				
Received	2109 (65.9)	66 (3.1)	1	1
Not received	1086 (33.9)	182 (16.8)	5.36 (3.94, 7.28)	4.93 (3.61, 6.75)
Unknown/missing	6 (0.2)	0 (0)	-	-
**Stratum**				
Low	1123 (35.1)	59 (5.3)	0.57 (0.38, 0.84)	0.77 (0.49, 1.20)
Medium	1028 (32.1)	92 (9.0)	0.97 (0.68, 1.39)	1.45 (0.94, 2.23)
High	1050 (32.8)	97 (9.2)	1	1
**Age (in Years)**				
≥18 to ≤ 59	2607 (81.4)	172 (6.6)	0.52 (0.40, 0.66)	0.55 (0.42, 0.71)
≥60	594 (18.6)	76 (12.8)	1	1
**Gender**				
Male	1975 (61.7)	178 (9.0)	1.58 (1.23, 2.02)	1.38 (1.05, 1.80)
Female	1226 (38.3)	70 (5.7)	1	1
**Education**				
Cannot read or write	752 (23.5)	68 (9.0)	1.72 (1.10, 2.68)	1.28 (0.83, 1.96)
Literate without formal schooling	408 (12.8)	29 (7.1)	1.35 (0.85, 2.16)	1.25 (0.80, 1.94)
Any formal schooling	1604 (50.1)	128 (8.0)	1.52 (1.03, 2.24)	1.28 (0.87, 1.88)
Any college education	437 (13.7)	23 (5.3)	1	1
**Occupation**^†^				
Self-employed	424 (13.3)	40 (9.4)	1.21 (0.85, 1.71)	-
Employed	1396 (43.6)	109 (7.8)	1	-
Economically inactive	1381 (43.1)	99 (7.2)	0.92 (0.72, 1.17)	-
**Monthly household Income before TB (quintiles)**^@^				
1st quintile	643 (20.1)	53 (8.2)	1.25 (0.81, 1.94)	1.16 (0.74, 1.82)
2nd quintile	703 (22.0)	57 (8.1)	1.23 (0.82, 1.84)	1.21 (0.83, 1.77)
3rd quintile	601 (18.8)	42 (7.0)	1.06 (0.75, 1.51)	1.02 (0.72, 1.44)
4th quintile	601 (18.8)	52 (8.7)	1.31 (0.90, 1.92)	1.42 (0.99, 2.05)
5th quintile	637 (19.9)	42 (6.6)	1	1
Unknown/Missing	16 (0.5)	2 (12.5)	-	-
**Notifying sector**				
Public	2911 (90.9)	231 (7.9)	1	-
Private	290 (9.1)	17 (5.9)	0.74 (0.39, 1.40)	-
**Site of disease**^$^				
Extra pulmonary	763 (23.8)	32 (4.2)	0.51 (0.31, 0.85)	0.78 (0.49, 1.26)
Pulmonary	2421 (75.6)	199 (8.2)	1	1
Unknown/missing	17 (0.5)	17 (100)	-	-
**Drug type**^**^**^				
DSTB	3128 (97.7)	231 (7.4)	0.33 (0.22, 0.51)	0.38 (0.24, 0.59)
DRTB	72 (2.3)	16 (22.2)	1	1
Unknown/missing	1 (0.03)	1 (100)	-	-
**HIV**				
Reactive	32 (1.0)	3 (9.4)	-	-
Non-reactive	3116 (97.3)	234 (7.5)	-	-
Unknown/missing	53 (1.7)	11 (20.8)	-	-
**Diabetes**				
Diabetic	307 (9.6)	33 (10.8)	1.61 (1.12, 2.31)	1.35 (0.93, 1.97)
Non-diabetic	2782 (86.9)	186 (6.7)	1	1
Unknown/missing	112 (3.5)	29 (25.9)	3.87 (2.56, 5.86)	2.58 (1.58, 4.22)

^**†**^Self-employed (business/farm/shop); employed (regular employee government/regular employee private/temporary employee (government and private)/skilled worker/daily wage earner); economically inactive (unemployed/homemaker/retired/pensioner/student).

*For adjusted PR, *p* value of 0.2 is considered for statistical significance, 1 – reference category.

@N = 3185; $N = 3184; ^N = 3200.

Abbreviations: DSTB, Drug-sensitive tuberculosis; DRTB, Drug-resistant tuberculosis; HIV, Human Immunodeficiency Virus.

## Discussion

In our cross-sectional study of 3201 PwTB in India, 92.7% had been credited at least one NPY instalment of whom 65.9% were aware of receipt of the benefit. The median delay in receipt of first benefit was around three months. One-fourth of the PwTB had received the benefit after declaration of treatment outcome. Most of the recipients had utilised the benefit wholly or partially for nutrition. The PwTB who did not receive NPY were more likely to experience unfavourable treatment outcomes.

The coverage of NPY was high compared to the earlier evaluations [15,18,23,26–28] possibly due to stronger political commitment to End TB, rectification of technical hiccups reported in early stages, better training of the implementing staff, and closer monitoring of NPY performance at district and state levels. The proportion of non-NPY receipt was highest among PwTB in low TB score stratum (15.3%) compared to the other strata. This may be reflective of the overall programmatic performance in the states in this stratum of which the TB score is an indicator. Previous studies conducted in low TB score states had also reported low coverage of 52.6% [31], and 10% [29] in Delhi. Other reasons for the difference in performance between the states could be due to state or district specific challenges such as being home to high proportion of migrants [31,32] and overburdening of staffs [28] or specific innovations that could have improved performance in some states.

Similar to our study, lack of bank account was reported as a major factor for the non-receipt of NPY in the previous studies [15,23,28,31]. This is because of lack of essential documents to open bank account [27], non-functional bank account [26,31], and migrant population [28]. Opening of bank account is still challenging for some PwTB especially among migrant population [28,31] and elderly despite initiatives like Pradhan Mantri Jan-Dhan Yojana (PMJDY) scheme [33], an initiative by the Government of India enabling people to open bank accounts with zero balance.

One-third of the PwTB were not aware of the receipt of the benefit. This could have been due to poor digital literacy [23], geographical access difficulties making it difficult to visit banks or ATMs, lack of counselling of the PwTB on benefits of better nutrition and provisions under NPY by the healthcare worker [34,35]. Unaware of receiving the benefit, the PwTB are also unable to utilize the benefit for nutrition.

A quarter of the PwTB had received their first instalment after their treatment outcome was declared. Similar long delays have been reported in Delhi with more than half of the PwTB receiving the first instalment during fourth and fifth month of treatment [31] and Gujarat where 34.4% PwTB received the first instalment after 6 months of treatment initiation [23]. Possible reasons for this delay could be due to the fact that bank accounts in certain rural or co-operative banks have difficulty in processing electronic transfers, re-initiating transfer after correcting bank details [28], merging of banks [26], complex formats of *Ni-Kshay* platform [36], technical issues due to updation and improvement of *Ni-Kshay* and PFMS portal [37], mismatch or rejection of bank details, unwillingness of PwTB to provide bank details [15,28], and more than one person using the same bank account [28], and the possible overload of NTEP staff [31,37]. Such delay precludes the timely utilization of the benefit by the PwTB for their nutrition.

Though received late, 88.8% of the PwTB reported fully utilising the NPY benefit for their nutrition. Similar to our results, earlier studies reported, 76% [34] and 64% [24] of the PwTB had utilised the benefits for their nutritional requirements. The possible reasons for the remaining 10.2% of PwTB not using the benefit for nutritional requirements could be the delay in receipt, lack of awareness about the purpose of the NPY benefit, and prioritising other household needs over their nutrition. There is a need for proper counselling of PwTB receiving treatment for tuberculosis about the importance of nutrition in their treatment. As reported in a previous study [38], the PwTB felt the monetary incentive is insufficient to meet their nutritional requirements. MoHFW recommends 40 Kcal/kg/day and 1.2 to 1.5 g/kg/day for an adult with BMI 21 kg/m^2^ [6]. Thus, an adult will require approximately 300 Kcal and 30 g protein in addition to his daily requirement of calories and proteins, which may cost a minimum of INR 750 to 1000 per month (US$ 9.1 to 12.1).

Earlier studies have also reported that PwTB who had not received the instalment were more likely to experience unfavourable treatment outcome such as loss of follow-up and death during treatment [15,39]. Timely receipt of instalment enables the PwTB to procure additional nutrition [24] in the form of protein-rich foods, supplements which improve tolerance to medication [40] and reduce loss to follow-up [15]. The better nutrition boosts immunity, also influences the pharmacodynamics and pharmacokinetics of the anti-TB drugs improving bacteriological clearance [41]. Boccia et al. discuss the numerous pathways through which a cash transfer intervention may impact TB incidence and treatment outcome [42]. Our cross-sectional design allows us to only point to this possible association, which has to be confirmed in a prospective study design or in trial settings in future studies.

### Strengths and limitations

This is the first study conducted on a nationally representative sample to determine the coverage and utilization of NPY for nutrition. The variables on the generation of NPY benefits, status of credit, and dates of credit are auto-generated by the system leaving no room for errors due to manual entry or bias in analysis.

The anthropometric measures of PwTB are not recorded uniformly or in a standardized manner in *Ni-kshay* at the initiation of treatment. Hence, we could not assess the change in the nutritional status of PwTB through the course of anti-TB treatment. Objective verification of receipt of NPY benefit or its utilisation could not be done due to nonuniform availability of bank passbooks, or electronic messages of benefit credit or food bills among the PwTB interviewed.

## Conclusion

Majority of the PwTB in India receive NPY, and most of them utilise it for nutrition. However, one-third continue to experience long delays to receive the first NPY instalment.

### Recommendation

In order to reduce the delays in credit of instalment, we may increase the amount paid as first benefit and reduce the frequency of instalments to enable PwTB to utilise the benefit. The amount provided as benefit may be increased commensurate with the additional nutritional requirements recommended for PwTB. In areas where access to banking is still difficult and digital literacy is poor, we may consider alternate modes of credit of the NPY benefit or other modes of nutritional support through the program. Recording of height and weight of PwTB at diagnosis, at every follow-up visit and at treatment outcome assignment may be standardised to enable analysis determining effect of NPY benefit receipt on nutritional status and treatment outcome. To elicit the complex causal pathway between receipt of a cash benefit for nutritional support and TB treatment outcomes, future operational research may focus on novel designs and modelling exercises to address confounders and mediators.

## Supplementary Material

NPY_evaluation_group.docx

## Data sharing policy

The datasets used and analysed in the present study are available from the corresponding author upon reasonable request.
