# Correction

**DOI:** 10.1080/16549716.2024.2390310

**Published:** 2024-08-29

**Article title**: *Ni-kshay Poshan Yojana*: receipt and utilization among persons with TB notified under the National TB Elimination Program in India, 2022

**Authors**: Kathiresan Jeyashree, Jeromie W V Thangaraj, Devika Shanmugasundaram, G Sri Lakshmi Priya, Sumit Pandey, Venkateshprabhu Janagaraj, Prema Shanmugasundaram, Sabarinathan Ramasamy, Joshua Chadwick, Sivavallinathan Arunachalam, Rahul Sharma, Vaibhav Shah, Aniket Chowdhury, Swati Iyer, Raghuram Rao, Sanjay K Mattoo, Manoj V Murhekar and NPY Evaluation Group

**Journal**: *Global Health Action*

**Bibliometrics**: Volume 17, Number 1, 2363300

**DOI**: http://dx.doi.org/10.1080/16549716.2024.2363300

When this article was originally published, a contributor was included in error. This has now been corrected.

