# Supplementary material for: Ni-kshay Poshan Yojana: receipt and utilization among persons with TB notified under the National TB Elimination Program in India, 2022
Source: Glob Health Action. 2024 Jul 22;17(1):2363300. doi: 10.1080/16549716.2024.2363300 (PMC11265306; doi:10.1080/16549716.2024.2363300)
Supplement: NPY_evaluation_group.docx [file ZGHA_A_2363300_SM9951.docx]

**NPY evaluation group**

| **Name** | **Affiliation** | **Email ID** |
| --- | --- | --- |
| B K Mishra | State TB Cell, Patna, Government of Bihar | stobi@rntcp.org |
| B K Vashisht | State TB Cell, New Delhi, Government of Delhi | stodl@rntcp.org |
| T K Soni | State TB Cell, Gandhinagar, Government of Gujarat | stogu@rntcp.org |
| M Mawrie | State TB Cell, R.P. Chest hospital, Shillong, Government of Meghalaya | stomg@rntcp.org |
| Prasanta Kumar Hota | State TB Cell, Bhubaneswar, Government of Odisha | stoor@rntcp.org |
| Vinod Garg | State TB Cell, Jaipur, Government of Rajasthan | storj@rntcp.org |
| Asha Federick | State TB Cell, Chennai, Government of Tamil Nadu | stotn@rntcp.org |
| A Rajesham | State TB Cell, Hyderabad, Government of Telangana | stots@rntcp.org |
| Pankaj Singh | State TB Cell, Dehradun, Government of Uttarakhand | stour@rntcp.org |
| MD Ashraf Rizvi | District TB cell, Sadar hospital campus, Katihar, Government of Bihar | dtobikth@rntcp.org |
| Purushottam Kumar | District TB cell, Sadar Hospital Campus, Begusarai, Government of Bihar | dtobibgs@rntcp.org |
| Anil Kataria | District TB cell, Baba Saheb Ambedkar Hospital, New Delhi, Government of Delhi | dtodlbsa@rntcp.org |
| Sunaina Anand | District TB cell, Dr. Hedgewar Aarogya Sansthan, New Delhi, Government of Delhi | dtodlhcc@rntcp.org |
| Vishal Khanna | District TB cell, Lok Nayak Hospital, New Delhi, Government of Delhi | dtodllnc@rntcp.org |
| Khalid Umer Khayyam | District TB cell, National Institute of TB & Respiratory Diseases, New Delhi, Government of Delhi | dtodllrs@rntcp.org |
| Devendra Parmar | District TB cell, GMERS Medical College, Patan, Government of Gujarat | dtogupat@rntcp.org |
| D N Barot | District TB cell, Civil Hospital, Nadiad, Government of Gujarat | dtogukda@rntcp.org |
| Zankhana Vasava | District TB cell, Civil Hospital, Narmada, Government of Gujarat | dtogunmd@rntcp.org |
| Easter Litha A Sangma | District TB cell, West Garo Hills, Government of Meghalaya | dtomgwgh@rntcp.org |
| E Kalwing | District TB cell, Ri Bhoi, Government of Meghalaya | dtomgrbh@rntcp.org |
| Bayarilin Shanpru | District TB cell, Shillong, Government of Meghalaya | dtomgekh@rntcp.org |
| Pravat Kumar Ojha | District TB cell, Deogarh, Government of Odisha | dtoordgr@rntcp.org |
| Biswabihari Mohanty | District TB cell, Angul, Government of Odisha | dtooragl@rntcp.org |
| Niharendra Panda | District TB cell, Kalahandi, Government of Odisha | dtoorklh@rntcp.org |
| Gunjan Khungar | District TB cell, Sri Ganganagar, Government of Rajasthan | dtorjggn@rntcp.org |
| Sudhir Sharma | District TB cell, Jaipur, Government of Rajasthan | dtorjjpr@rntcp.org |
| Kuldeep Meena | District TB cell, Bundi, Government of Rajasthan | dtorjbdi@rntcp.org |
| K Jayasree | District TB cell, Vellore, Government of Tamil Nadu | dtotnvlr@rntcp.org |
| M Sankari | District TB cell, Pudukkottai, Government of Tamil Nadu | dtotnpdk@rntcp.org |
| D Dheenadayal | District TB cell, Tiruppur, Government of Tamil Nadu | dtotntup@rntcp.org |
| M Srikanth. | District TB cell, Adilabad, Government of Telangana | dtotsadb@rntcp.org |
| K Kalyan Chakravarthi | District TB cell, Nalgonda, Government of Telangana | dtotsngd@rntcp.org |
| Vimal Gusain | District TB cell, Rudraprayag, Government of Uttarakhand | dtourrdp@rntcp.org |
| Ramesh Kunwar | District TB cell, Pauri, Government of Uttarakhand | dtourgrw@rntcp.org |
| Rajesh Dhakhariyal | District TB cell, Haldwani, Government of Uttarakhand | dtournnt@rntcp.org |
| R K Singh | District TB cell, Haridwar, Government of Uttarakhand | dtourhrd@rntcp.org |
